# *Leishmania tarentolae* as Potential Live Vaccine Co-Expressing Distinct Salivary Gland Proteins Against Experimental Cutaneous Leishmaniasis in BALB/c Mice Model

**DOI:** 10.3389/fimmu.2022.895234

**Published:** 2022-06-10

**Authors:** Mahya Sadat Lajevardi, Elham Gholami, Tahereh Taheri, Hamzeh Sarvnaz, Sima Habibzadeh, Negar Seyed, Yousef Mortazavi, Sima Rafati

**Affiliations:** ^1^Department of Medical Biotechnology, School of Medicine, Zanjan University of Medical Sciences, Zanjan, Iran; ^2^Department of Immunotherapy and Leishmania Vaccine Research, Pasteur Institute of Iran, Tehran, Iran

**Keywords:** cutaneous leishmaniasis, live vaccine, sand fly, salivary proteins, *Leishmania major*, *Leishmania tropica*, *Leishmania tarentolae*

## Abstract

Leishmaniasis is a neglected vector-borne disease caused by *Leishmania* parasites transmitted through the infected sand flies bite. Current treatments are limited, partly due to their high cost and significant adverse effects, and no human vaccine is yet available. Sand flies saliva has been examined for their potential application as an anti-*Leishmania* vaccine. The salivary protein, PpSP15, was the first protective vaccine candidate against *L. major*. Additionally, PsSP9 was already introduced as a highly immunogenic salivary protein against *L. tropica*. Herein, we aimed to develop an effective multivalent live vaccine to control Cutaneous Leishmaniasis induced by two main species, *L. major* and *L. tropica*. Hence, the two above-mentioned salivary proteins using T2A linker were incorporated inside the *L. tarentolae* genome as a safe live vector. Then, the immunogenicity and protective effects of recombinant *L. tarentolae* co-expressing PpSP15 and PsSP9 were evaluated in pre-treated BALB/c mice with CpG against *L. major* and *L. tropica*. Following the cytokine assays, parasite burden and antibody assessment at different time-points at pre and post-infection, promising protective Th1 immunity was obtained in vaccinated mice with recombinant *L. tarentolae* co-expressing PpSP15 and PsSP9. This is the first study demonstrating the potency of a safe live vaccine based on the combination of different salivary proteins against the infectious challenge with two different species of *Leishmania*.

## Introduction

Leishmaniasis is a neglected vector-borne disease caused by protozoan parasites of the *Leishmania* genus. Cutaneous Leishmaniasis (CL) with the highest incidence rate, is one of the major clinical manifestations of the disease and is mainly characterized by self-healing ulcerative lesions (1). Annually, almost one million new CL cases are recognized. In 2020, more than 85% of the cases belonged to 10 countries including Iran, where about 20,000 new cases occur per year (2). Two different forms of CL including zoonotic cutaneous leishmaniasis (ZCL) and anthroponotic cutaneous leishmaniasis (ACL) occur in Iran (3). The causative agent of ZCL which is observed in the rural area is *Leishmania major* (*L. major*) while ACL is related to urban areas and caused by *Leishmania tropica* (*L. tropica*) (4–6). The parasites are transmitted to the host through the bites of infected sand flies (the Phlebotomus species in the Old World and the Lutzomyia species in the New World) in the context of sand fly saliva (7, 8). Saliva contains bioactive proteins that facilitate blood-feeding by vectors (9). Evolutionarily, parasites have evolved to take advantage of this blood-feeding process and nowadays, there is strong evidence that saliva promotes *Leishmania* infection in vertebrate host (10).

Interestingly, pre-exposure to the uninfected sand fly bite of several species or immunization with certain salivary proteins induces protective Th1 adaptive responses in rodents against subsequent infection with some *Leishmania* species (11–15). It has been postulated that the saliva-mediated protective anti-*Leishmania* immunity most probably correlates with the induction of an early phase Th1 response which prevents permissive phagocyte formation and effectively directs towards disease control (16, 17). This has drawn much attraction toward saliva-based vaccines composed of immunostimulatory components of saliva alone or along with parasite antigens. In this respect, SP15 protein from *Phlebotomus papatasi* (*Ph. Papatasi*) was among the first vaccine candidates introduced to control *L. major* infection by different formulations (18–21). Today, several salivary proteins from different sand fly species are identified as potential candidates but less is known about *Ph. sergenti*, the vector for *L. tropica* transmission in the old world. Recently, it has been demonstrated that mice immunization with the whole *Ph. sergenti* saliva does not protect against the intradermal challenge of *L. tropica* plus *Ph. sergenti* salivary gland homogenate (SGH). Instead, a potent Th1 driving component among different *Ph. sergenti* saliva constituents with protective potential against *L. tropica* infection (PsSP9) was identified (22).

Various vaccination strategies notably live vaccines are now under intensive investigation to meet the requirements for an effective vaccine against leishmaniasis (23, 24). Among live vaccines, non-pathogenic candidates have extraordinary benefits which appropriately meet safety issues. In this approach, live *Leishmania tarentolae* (a lizard hosted parasite) came into focus as surrogate for leishmanization, years ago after it was distinguished as non-pathogenic in mice (25). Later on, owing to advancements in parasite recombination protocols and the availability of plasmid vectors to generate stable transfectants, *Leishmania tarentolae* was manipulated to deliver overexpressed antigens either from parasite or even sand fly-related proteins (19, 20, 26). Of note, non-pathogenic *L. tarentolae* has significant genetic similarities to pathogenic species (27). From this perspective, these parasites are an excellent choice in vaccine design that deliver both *Leishmania* antigens and intentionally inserted non-*Leishmania* antigens (for example saliva proteins) to the host while inducing a Th1-mediated response (14).

Respecting the importance of salivary components and advantaging a potential Th1-inducing delivery system, recombinant *L. tarentolae* co-expressing full PpSP15 and PsSP9 were generated in this study with the aim of dual vaccine design against both *L. major* and *L. tropica* infection. T2A linker (a virus-derived self-cleavable peptide) was inserted between the genes encoding vaccine candidates to produce proteins in separate secretory forms. Then, the immunogenicity and protective efficacy of this novel live vaccine along with a new CpG regime as the adjuvant was evaluated in BALB/c mice model. The results indicated that dual vaccine formulation using salivary components is a hope and not hype and can improve vaccine availability in endemic areas where different sand fly species are frequent and transmit different parasites.

## Materials and Methods

### Ethics Statement

In this study, female BALB/c mice (6–8 weeks old) were obtained from the Pasture Institute of Iran and kept under standard conditions of the animal facility in Pasture Institute of Iran (a constant 12h light-dark cycles, relative humidity 50–60%, and free access to normal rodent chow diet). All animal experiments were performed according to the animal care guideline approved by Institutional Animal Care and Research Advisory Committee of Pasteur Institute of Iran (ethical code: IR.RII.REC.1394.0201.6417, dated 2015) in full compliance with the Specific National Ethical Guidelines for Biomedical Research (MOHME-2005).

### Reagents

All needed solutions were prepared by pyrogenic deionized water (Milli-Q System, Millipore, France). Sodium dodecyl sulfate (SDS), Ficoll-400, Tris-base, Tris-HCL, and cell culture reagents including and Schneider′s Insect Medium, DMEM medium, HEPES, L-glutamine, adenosine, hemin were purchased from Sigma (Germany). Fetal Calf Sera (FCS), G418 were provided from Gibco (Life Technologies GmbH, Germany). Bovine Serum Albumin (BSA) and Diaminobenzidine (DAB) powder were supplied by Merck (Germany). All materials for gel electrophoresis, PCR, and enzymatic digestion were prepared by Roche Applied Sciences (Germany). Specific primers for target genes amplification were synthesized by metabion (Germany). Cytokine kits (IFN-γ, IL-4, IL-10 and IL-17) were purchased from DuoSet R & D (USA). Quanti Nova SYBR Green Master Mix, Anti-His Tag Antibody, RNeasy kit, QuantiTect Reverse Transcription Kit, and GF-1 Tissue DNA Extraction Kit were purchased from QIAGEN (Germany) and Vivantis (Vivantis Technologies, Malaysia), respectively. Pierce™ BCA^®^ (Bicinchoninic Acid) protein assay kit was provided by Thermo Fisher Scientific (USA). Horseradish peroxidase-conjugated goat anti-mouse IgG1 and IgG2 were provided by Southern Biotech (Canada). Peroxidase Substrate for ELISA systems was obtained from KPL (ABTS, USA). CpG oligodeoxynucleotide (ODN), known as CpG ODN-1826 (6326.7 g/mol, MW), with the sequence 5′-TCCATGACGTTCCTGACGTT-3′, was prepared from Microsynth (Balgach, Switzerland). pLEXSY-neo2 vector was obtained from Jena bioscience (Germany).

### *L. tarentolae* PpSP15-T2A-PsSP9 Generation by Stable Transfection

The PpSP15-T2A-PsSP9 construct was synthesized by Thermo Fisher Scientific Company (USA) and delivered in the pMA-RQ plasmid (PpSP15-T2A-PsSP9 sequence arrangement was selected among different combinations by bioinformatic analysis based on RNA secondary structure and protein stability but data is not shown here). Following sequence confirmation, the PpSP15-T2A-PsSP9 fragment was digested out by *Bgl*II and *Nhe*I restriction enzymes and sub-cloned into pLEXSY-neo2 vector (typically used as an integrative vector for stable genome transfection in *L. tarentolae*) pre-digested by the same enzymes. According to preset transfection protocols (28), 4×10^7^ log-phase *L. tarentolae* (Tar II ATCC 30267) parasites were re-suspended in 300 µl of ice-cold electroporation buffer (0.7mM Na_2_HPO_4_, 137mM NaCl, 5mM KCl, 21mM HEPES, 6mM glucose; pH 7.5) and mixed with 10 µg of linearized pLEXSY- PpSP15-T2A-PsSP9 by *Swa*I enzyme (5989 bp). After 10 min of incubation on ice, cells were electroporated at 450 V and 500 mF (Bio-Rad Gene Pulser Ecell device (Germany)). The electroporated parasites were cultured in supplemented antibiotic-free M199 (5% FBS) medium at 26°C for 24 hr. Antibiotic-resistant parasites were then selected on a semi-solid medium containing M199, 50% Noble agar, and 50 µg/ml G418.

### Confirming Recombinant Parasites at DNA, RNA, and Protein Levels

Stable recombinant *L. tarentolae* parasites co-expressing PpSP15 and PsSP9 proteins were further confirmed at the DNA, RNA, and protein levels by diagnostic PCR, reverse-transcription PCR (RT-PCR), and Western blot, respectively. Diagnostic PCR was performed using the transfectants’ genomic DNA and 5’SSU specific forward (F3001 5’-GATCTGGTTGATTCTGCCAGTAG-3’) and reverse (A1715 5-TATTCGTTGTCAGATGGCGCAC-3) primers to generate a 1-kb fragment. F3001 primer targets upstream of the 5’SSU region on *L. tarentolae* genome and A1715 primer binds to the pLEXSY backbone(downstream of 5'SSU region and upstream of PpSP15-T2A-PsSP9 construct). For RT-PCR, total RNA from 2×10^8^ recombinant parasites was extracted using RNeasy kit and cDNA was synthesized according to the QuantiTect Reverse Transcription Kit instructions. Targets were separately amplified using specific primers for a part of each individual gene (PpSP15- forward: 5’-ACGCCTCCAAAATGGCTT-3’ and reverse: 5’-TTGACCTTTTTGGCGCACTC-3, PsSP9- forward: 5’- CATTGTGAATACCGTGCTTACG-3’ and reverse 5’-GAGTTTGTTCGAGTGCTTGG-3’). At the protein level, the expression of PpSP15 and PsSP9 by recombinant *L. tarentolae* was confirmed by Western blot analysis. Briefly, the concentrated supernatant of recombinant *L. tarentolae* at stationary phase was mixed with SDS-PAGE sample buffer (4.5mMTris–HCl, pH 6.8, 2%, w/v SDS, 5%, v/v 2-mercaptoethanol, 10%, v/v glycerol, 0.05%, w/v bromophenol blue) and run on 17.5% SDS-PAGE polyacrylamide gel. In the next step, proteins were transferred to the nitrocellulose membrane and free binding sites were blocked by incubation of the membrane with a blocking solution (TBS with 2.5% BSA and 0.05% Tween 20) overnight. Then, the primary Anti-His Tag Antibody (1/2000) was added to the membrane at room temperature. After 2 hr, HRP-conjugated goat anti-mouse IgG (1/7000) as the secondary antibody was used. Following incubation for 2 hr and three washes, Diaminobenzidine tetrahydrochloride (DAB) was applied as the substrate for visualization.

### Parasite Culture and Antigen Preparation

BALB/c mice already infected with *L. major* (MRHO/IR/75/ER) and *L. tropica* (MOHM/IR/09/Khamesipour-Mashhad) were sacrificed for parasite isolation. Isolated lymph nodes were then incubated in Schneider medium supplemented with 10% FCS and 100μg/ml of Gentamicin at 26°C for *Leishmania* promastigote propagation. The infective-stage metacyclic parasites at stationary-phase were separated on Ficoll 400 gradient and were collected for the subsequent infectious challenge. For *in vitro* stimulation assays, the stationary-phase promastigotes of *L. tarentolae* PpSP15-T2A-PsSP9, *L. major*, and *L. tropica* parasites were repeatedly freeze-thawed to prepare F/T antigen. Briefly, promastigotes were collected from the cultures and washed with PBS (8 mM Na_2_HPO_4_, 1.75 mM KH_2_PO_4_, 0.25 mMKCl, and 137 mM NaCl). 2×10^8^ parasites/ml were then embedded in liquid nitrogen for a few seconds and immediately thawed in 37°C water-bath. This process was repeated until no intact parasite was discriminated under the light microscope. The protein concentration was then quantified by BCA method (29).

### Immunization and Infectious Challenge

Immunization was carried out for three female BALB/c mice groups (n= 28 per group) three times in 3-weeks intervals (**Figure S1**). Initially, all groups received CpG ODNs (45μg/mice) as an adjuvant intraperitoneally. After 3hr, groups were immunized with 2×10^7^ *L. tarentolae* PpSP15-T2A-PsSP9 (G1), 2×10^7^ wild-type *L. tarentolae* (G2) or PBS (G3) accordingly (**Table 1**). All mice were immunized subcutaneously (s.c.) at the left hind footpad in a total volume of 50 μl. Three weeks after the last immunization, each group was divided into two separate boxes for the infectious challenge either by *L. major* or *L. tropica* metacyclic promastigotes along with pertinent salivary gland homogenate (SGH, 0.5 pair/mouse) derived from *Ph*. *papatasi* and *Ph. sergenti* respectively (provided by Dr. Shaden Kamhawi and Dr. Jesus G. Valenzuela, NIH, USA). Immunized BALB/c mice were challenged s.c with either 2×10^5^ *L. major* plus *Ph*. *papatasi* SGH or 10^7^ *L. tropica* plus *Ph. sergenti* SGH at the right hind footpad (**Table 1**).

**Table 1:** Vaccination regimens in BALB/c mice groups.

Bch groups	Prime	Boost	Boost	Ach groups	Challenge
**G1**	CpG *+ L. tarentolae* PpSP15-T2A-PsSP9	CpG *+ L. tarentolae* PpSP15-T2A-PsSP9	CpG *+ L. tarentolae* PpSP15-T2A-PsSP9	**G1Lm**	*L. major +* SGH *_Ph. papatasi_*
				**G1Lt**	*L. tropica +* SGH *_Ph. sergenti_*
**G2**	CpG *+ L. tarentolae*	CpG *+ L. tarentolae*	CpG *+ L. tarentolae*	**G2Lm**	*L. major +* SGH *_Ph. papatasi_*
				**G2Lt**	*L. tropica +* SGH *_Ph. sergenti_*
**G3**	CpG + PBS	CpG + PBS	CpG + PBS	**G3Lm**	*L. major +* SGH *_Ph. papatasi_*
				**G3Lt**	*L. tropica +* SGH *_Ph. sergenti_*

Before challenge (Bch), all groups were shown as G1 (CpG + L. tarentolae PpSP15-T2A-PsSP9) for the vaccinated group and G2 (CpG + L. tarentolae) and G3 (CpG + PBS) as control groups. Following infectious challenge (Ach) with L. major +SGH _Ph. papatasi_ and L. tropica + SGH _Ph. sergenti_ each group was shown as G1Lm, G2Lm, G3Lm and G1Lt, G2Lt, G3Lt, respectively.

### Footpad Thickness Measurement

Following *L. major* infection, footpad thickness was weekly monitored with a digital caliper from week two post-challenge until week eleven for 10 weeks. For this, the mice were restricted and the thickness was measured as the diameter of the swelling site by fixing the caliper on the back and forth sides of the footpad. The uninoculated collateral footpad was simultaneously monitored as control.

### Lymph Node Parasite Load Determination by Real-Time PCR

Parasite load in popliteal lymph nodes (pLNs) was estimated at 7^th^ and 11^th^ week post challenge (7^th^WAC and 11^th^WAC) by quantitative Real-Time PCR. Four mice per group were sacrificed at each time point. Genomic DNA (gDNA) from homogenized lymph nodes was extracted using GF-1 Tissue DNA Extraction Kit according to the manufacturer’s instruction. DNA purity and concentration were assessed by Nanodrop (ND-1000). According to preset protocols (19, 22), two specific primer sets were used to target a part of kinetoplastid minicircle regions of parasites (*L*. *tropica*: KDNAF, 5’-GGGTAGGGGCGTTCTGC-3’ and KDNAR, 5’-TACACCAACCCCCAGTTTGC-3, *L. major:* RV1F, 5’-CTTTTCTGGTCCCGCGGGTAGG-3’and RV2R, 5’-CCACCTGGCCTATTTTACACCA-3’). The absolute copy number of parasites was measured using the StepOnePlus Real-Time PCR device and software (Applied Biosystems). In parallel, mouse glyceraldehyde 3-phosphate dehydrogenase (GAPDH) as a reference gene was quantified with GAPDHF, 5’-CGTCCCGTAGACAAAATGGT-3’ forward and GAPDHR, 5’-TTGATGGCAACAATCTTCAC-3’ reverse primers to normalize the parasite load (30). Parasite load was then determined using the standard curve generated by 10 fold dilutions of gDNA extracted from each parasite in a range of 10^8^−10^1^ parasite per reaction (extracted gDNA from *L. major* and *L. tropica* was used to measure the load of *L. major* and *L. tropica*, respectively). The amount of used gDNA per each PCR reaction was 20 and 80 nanograms for *L. major* and *L. tropica*, respectively. Likewise, the standard curve for the reference gene was obtained using serial dilutions of naive mouse gDNA (isolated from LN) in the range of 2.7 ×10^4^ to 1 ×10^2^ DNA copies per reaction. The total volume of each PCR reaction containing 5 μl of 2x Quanti Nova SYBR Green Master Mix, 500 nM of each forward and reverse primers, and 1 μl carboxyrhodamine or ROX (passive dye) was 10 μL. The PCR amplification was set on the following program: 95°C for 20 s, 40 cycles consisting of 95°C for 3s and 60°C for 30 s. PCR reactions were all in duplicate and the efficiencies of target and reference genes were 100%. The normalized parasite load was calculated using the formula: [(parasite numbers)/(GAPDH copy numbers/2)] and plotted as the number of *Leishmania* parasites per cell.

### Analysis of Cytokine Production Pre- and Post-Challenge

Cytokine production by vaccinated and control groups was measured at pre-and two time points post-challenge (7^th^WAC and 11^th^WAC). To this end, three or four mice per group were sacrificed at each time point. Then, the isolated spleens were homogenized after erythrocyte lysis by ACK lyses buffer (0.15MNH_4_Cl, 1mM KHCO_3_, and 0.1 mM Na_2_EDTA). Splenocytes were washed and then re-suspended in complete phenol red-free DMEM medium containing 10% FCS, 1% L-glutamine, 1% HEPES, 0.1% 2ME, and 0.1% gentamicin. 3.5×10^6^ cells were plated in 48 well plates and were re-stimulated by different antigens including *L. tarentolae* PpSP15-T2A-PsSP9 F/T (20 μg/ml) for pre-challenge, *L*. *major* F/T (20 μg/ml), and *L*. *tropica* F/T (20 μg/ml) for post-challenge. In all experiments, Concanavalin A (ConA, 5 μg/ml) and culture media were used as positive and negative controls, respectively. The incubation time (37°C in a humidified atmosphere with 5% CO_2_) was 72 hr for IL-4 and IL-17, but 120 hr for the other two cytokines, IFN-γ and IL-10. The cytokines concentrations in the cells culture supernatants were measured by sandwich ELISA kits according to the manufacturer’s protocols. The minimum limit of detection for IFN-γ, IL-4, IL-10, and IL-17 was 2, 3, 4, and 5 pg/ml, respectively.

### Nitric Oxide Measurement

The Nitric oxide (NO) production was also measured at 7^th^ and 11^th^ weeks after the infectious challenge. Following re-stimulation of mice splenocytes with the specific antigen including *L. major* F/T (20 μg/ml) or *L. tropica* F/T (20 μg/ml) for 120h, Nitrite concentration was measured in the cells supernatant by Griess reagent containing naphthyl ethylenediamine (NED) dihydrochloride (0.1 N) and 1% sulfanilamide in 5%H_3_PO_4_ mixed together in equal volumes before use. For Nitrite measurement, 100 μL of each sample was mixed with an equal amount of Griess reagent and incubated for 10 min at RT. The absorbance was determined at 540 nm. Different concentrations (0–300μM) of sodium nitrite (in phenol red-free DMEM) as standards were applied to plot the standard curve (31).

### Determination of Antibody Level

Before and 11 weeks after the infectious challenge, BALB/c mice were bled from retro-orbital sinus to determine antibody responses. The mice sera were analyzed using ELISA for specific IgG1 and IgG2a subclasses levels against F/T lysates of *L. tarentolae* PpSP15-T2A-PsSP9, *L. major*, and *L. tropica* (28). In brief, 96-well ELISA plates were coated with 10μg/mL of the above-mentioned antigens in bicarbonate buffer (Na_2_CO_3_ 0.02 M, NaHCO_3_ 0.45 M, pH 9.6) overnight at 4°C. Following three washes (PBS + 0.05% Tween 20), wells were blocked with 100 μl of 1% BSA in PBS for 2hr at RT to avoid nonspecific bindings. Then, 100 μl of diluted sera (1/150) in PBS containing 0.05% Tween 20 and 1% BSA were added and micro-plates were incubated for 2 hr at 37°C. Next, 100 μL diluted peroxidase-conjugated goat anti-mouse IgG1 (1:5000) and IgG2a (1:3000) antibodies were added to each pertinent well. Following incubation (2 hr-37°C) and washing, peroxidase substrate KPL ABTS (100 μL) was used for dye development in each well. The enzymatic reactions were stopped by 1% SDS and the absorbance was measured at 405 nm along with reference (630 nm).

### Statistical Analysis

GraphPad Prism 7.0 (GraphPad, CA, USA) was utilized for statistical analyses. Based on Shapiro-Wilk test and data passing normality, Student’s t-test was used for data analysis. *P*-values below 0.05 (p < 0.05) were considered statistically significant.

## Results

### Generation of Recombinant *L. tarentolae* Expressing PpSP15 and PsSP9 Salivary Proteins

**Figure 1:**
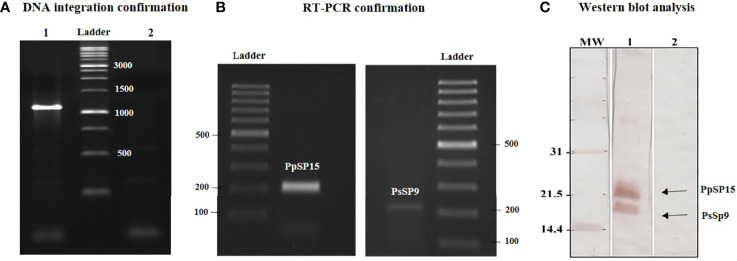
Generation of recombinant parasite (*L. tarentolae* PpSP15-T2A-PsSP9). **(A)** PCR amplification of the 1 kb band confirmed the integration of pLEXSY-PpSP15-T2A-PsSP9 into the parasite genome (lane 1). Lane 2 represents wild-type *L. tarentolae* as a negative control. **(B)** RT-PCR amplification of PpSP15 (left image) and PsSP9 (right image) confirmed recombination at the RNA level. **(C)** Western blot analysis for evaluating gene expression in recombinant *L. tarentolae* PpSP15-T2A-PsSP9. Lane 1 indicates proteins expression in recombinant *L. tarentolae* PpSP15-T2A-PsSP9. Lane 2 is wild-type *L. tarentolae* as a negative control.

Recombinant *L. tarentolae* stably expressing two different sand fly salivary proteins (PpSP15 and PsSP9) was generated by introducing the linearized pLEXSY-PpSP15-T2A-PsSP9 (5989 bp) into the 18S rRNA locus of *L. tarentolae* genome. It is worth mentioning that the T2A peptide is a self-cleavage linker which can lead to producing its upstream and downstream proteins separately. The integration of the expression cassette in transgenic *L. tarentolae* was initially confirmed at the DNA level by amplification of a 1-kb fragment (**Figure 1A**). Moreover, the expression of the salivary proteins was confirmed by both RT-PCR and Western blot. Each constituent gene of the PpSP15-T2A-PsSP9 cassette was amplified after cDNA preparation by specific primers (**Figure 1B**). At the protein level, two separate immunoreactive bands of approximately 15 kDa (PsSP9) and 18 kDa (PpSP15-T2A) in size were detected in Western blot using an anti-His-tag antibody (**Figure 1C**). Furthermore, non-cleaved 36 kDa protein was not detected; an indicator of full dissociation of the fusion into constituent proteins owing to T2A related self-cleavage. Of note, in this study T2A peptide was applied for the first time in *L. tarentolae*, which, despite its viral origin, was able to well function.

### Pre-Challenge Immune Response of the Live Recombinant *L. tarentolae* PpSP15-T2A-PsSP9 + CpG Vaccinated Mice Was Well Polarized Toward Th1

**Figure 2:**
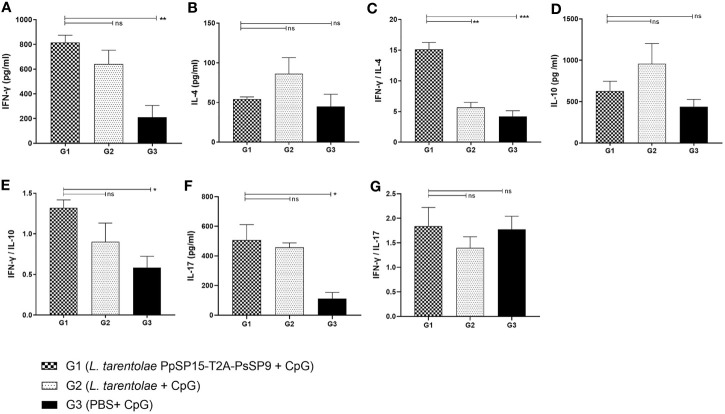
Before challenge analysis of cytokine production in vaccinated and control mice by ELISA. After the last immunization and prior to infectious challenge, all mice (n=3-4 per group) were sacrificed and single splenocytes from each individual BALB/c mouse were re-stimulated by *L. tarentolae* PpSP15-T2A-PsSP9 F/T antigen. **(A)** IFN-γ production, **(B)** IL-4 production, **(C)** IFN-γ/IL-4 ratio, **(D)** IL-10 production, **(E)** IFN-γ/IL-10 ratio, **(F)** IL-17 production, **(G)** IFN-γ/IL-17ratio. For all cytokines, the results are presented as mean ± SE (in pg/ml). Data are representative of two independent experiments. Student t-test was used for statistical analysis (*p< 0.05, **p < 0.01, ***p < 0.001, ns, non-significant).

After the last immunization and prior to infectious challenge with *L. major* and *L. tropica*, all mice groups were subjected to cellular and humoral immune response evaluation. Four effector cytokines including IFN-γ, IL-4, IL-10, and IL-17 were measured in the culture supernatant of mice splenocytes stimulated with *L. tarentolae* PpSP15-T2A-PsSP9 F/T antigen. It is well established that the higher level of IFN-γ (as the Th1 main cytokine) relative to IL-4 (as the Th2 main cytokine), IL-10 (as the anti-inflammatory cytokine), and IL-17 (as a neutrophil chemotactic cytokine) is the key factor to control the *Leishmania* infection. Therefore these cytokines were further evaluated in each differently vaccinated group. As shown in **Figure 2A**, higher levels of IFN-γ were detected in *L. tarentolae* PpSP15-T2A-PsSp9 vaccinated group (G1). Meanwhile, IL-4 was detected at a low amount in all three groups with no significant difference (**Figure 2B**). Given the importance of the IFN-γ/IL-4 ratio as Th1 response deviation indicator, the highest level was detected in G1 vaccinated group (*p*= 0.0017) (**Figure 2C**). On the other hand, the production of IL-10 as an indicator of immunoregulatory cytokine revealed no significant difference between groups (**Figure 2D**). Therefore, G1 indicated the highest level of IFN-γ/IL-10 ratio compared to G2 (*p*= 0.1721) and G3 (*p*= 0.0125) groups (**Figure 2E**). Furthermore, IL-17 production (**Figure 2F**) was remarkably induced in the vaccinated group (G1) however the ratio of IFN-γ/IL-17 in G1 group was non-significantly higher than G2 group (**Figure 2G**). This might indicate that IL -17 production is mainly controlled by CpG adjuvant rather than the SP15 or SP9 antigens (the IFN-γ/IL-17 ratio in PBS group was calculated high since both cytokines were produced in low amounts).

**Figure 3:**
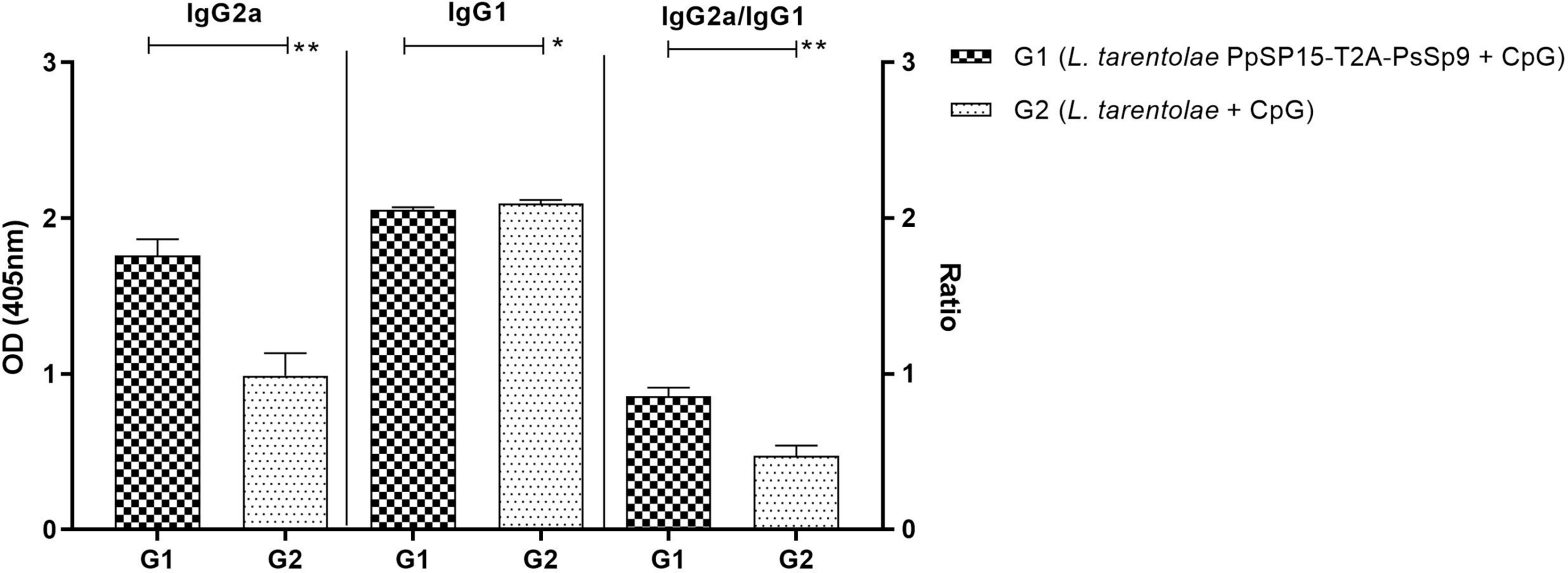
Analysis of antigen-specific antibody responses in vaccinated and control mice by ELISA before challenge. After the last immunization and prior to infectious challenge, four mice per group were bled from the venous sinus to collect sera. The level of IgG2a, IgG1, and IgG2a/IgG1 ratio were evaluated in response to *L. tarentolae* PpSP15-T2A-PsSP9 F/T antigen. Data are representative of two independent experiments. The results are presented as mean ± SE and student t-test was used for statistical analysis (*p < 0.05; **p < 0.01).

The humoral immune responses through the measurement of IgG subclasses were also determined for *L. tarentolae* PpSP15-T2A-PsSP9 vaccinated group (G1) and *L. tarentolae* wild-type control group (G2) in response to *L. tarentolae* PpSP15-T2A-PsSp9 F/T antigen. As known, the production of IgG2a is associated with Th1 immune responses in mice while IgG1 production is related to the Th2 profile. As indicated in **Figure 3**, the higher level of IgG2a and the lower level of IgG1 were significantly observed in the *L. tarentolae* PpSP15-T2A-PsSP9 vaccinated group (G1) compared to the control group (G2). Collectively, the above findings demonstrated a Th1-skewed response in vaccinated mice by *L. tarentolae* PpSP15-T2A-PsSP9 compared to the control groups (G2 and G3) relying on remarkable IFN-γ and IL-17 production versus negligible IL-4 and IL-10 in G1 vaccinated group. These results further indicated that sand fly protein-expressing *L. tarentolae* adjuvanted with CpG can manipulate the immune system in favor of Th1 response which is obviously the prerequisite of *Leishmania* infection control after infectious challenge.

### *L. major* Infectious Challenge Is Partially Tolerated in *L. tarentolae* PpSP15-T2A-PsSP9 Vaccinated BALB/c Mice

**Figure 4:**
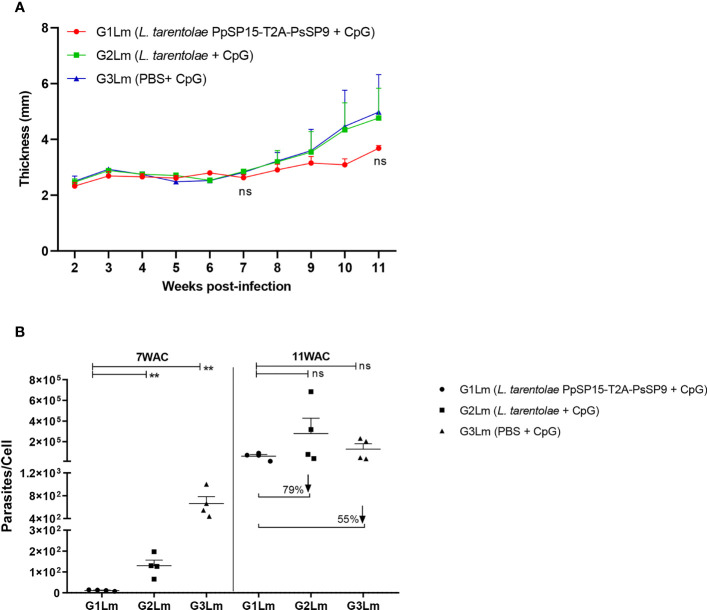
Assessment of footpad thickness and parasite load in vaccinated and control BALB/c mice infected with *L. major* +SGH. Pre-treated BALB/c mice (n=28 per group) with CpG were immunized at the left footpad with *L. tarentolae* PpSP15-T2A-PsSP9 (G1), *L. tarentolae* (G2), and PBS (G3). Three weeks after the last booster, mice were challenged with *L. major* metacyclic promastigotes plus *Ph. papatasi* SGH in the contralateral footpad. **(A)** The inflammation progression was monitored weekly by measuring footpad thickness. Data are representative of two independent experiments. Results are exhibited as the mean ± SE and student t-test was used for statistical analysis (*p*-value < 0.05 as significant, ns, non-significant). **(B)** The number of parasites per mouse lymph node cell was determined at weeks 7 and 11 post-infection by Real-Time PCR (n=4 mice per group). Results are exhibited as the mean ± SE. Data are representative of two independent experiments. Student t-test was used for statistical analysis (**p < 0.01; ns, non-significant). The arrows show the percentage reduction of parasite load in G1Lm in respect to control groups at week 11 after infectious challenge.

Three weeks following the last immunization, half of the BALB/c mice in each G1, G2, and G3 groups were challenged by *L. major* metacyclic promastigotes along with SGH of *Ph. papatasi* and denoted as G1Lm, G2Lm, and G3Lm (**Table 1**). Typically, the vaccine efficacy against *L. major* infection is evaluated by footpad thickness monitoring and parasite burden assessment. As indicated in (**Figure 4A**) weekly measurement of footpad thicknesses up to week 11 displayed a non-significant control on the thickness increase in mice immunized by *L. tarentolae* PpSP15-T2A-PsSP9 (G1Lm) compared to control groups (G2Lm and G3Lm). Meanwhile, quantitative RT-PCR at week 7 post-challenge revealed a significantly lower level of parasite number per lymph node cell in G1Lm group (mean ± SE= 11.5 ± 1.6) compared to G2Lm (mean ± SE= 129.9 ± 26.7) and G3Lm (mean ± SE= 662.4 ± 121.9) groups (**Figure 4B**). Later quantification at week 11 however indicated no significant difference between groups although the lowest parasite number per lymph node cell was detected in G1Lm (mean ± SE= 5.9×10^4^ ± 1.7×10^4^) versus G2Lm (mean ± SE= 28×10^4^ ± 1.5×10^4^) and G3Lm (mean ± SE= 13×10^4^ ± 5.1×10^4^). In other words, *L. tarentolae* PpSP15-T2A-PsSP9 plus CpG was able to reduce the parasite load in G1Lm by nearly 80% and 55% compared to G2Lm and G3Lm, respectively at later weeks (**Figure 4B**).

**Figure 5:**
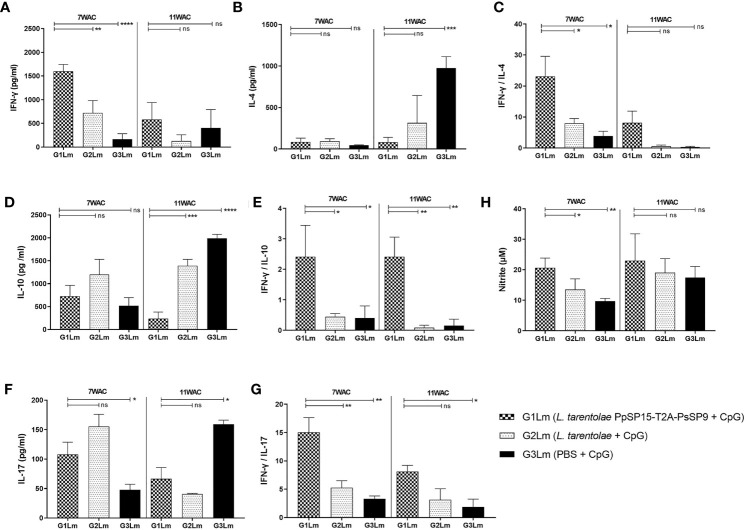
Analysis of cytokines production in vaccinated and control mice post-infection with *L. major*+SGH*_Ph. papatasi_*. Following 7^th^ and 11^th^ weeks after challenge, all mice (n=3-4 per group) were sacrificed and single splenocytes from each individual BALB/c mouse were re-stimulated by *L. major* F/T antigen. **(A)** IFN-γ production, **(B)** IL-4 production, **(C)** IFN-γ/IL-4 ratio, **(D)** IL-10 production, **(E)** IFN-γ/IL-10 ratio, **(F)** IL-17 production, **(G)** IFN-γ/IL-17 ratio, **(H)** Nitric oxide (NO) production. The results are presented as mean ± SE in pg/ml for cytokines or in µm for NO. Data are representative of two independent experiments. Student t-test was used for statistical analysis (*p < 0.05; **p < 0.01; ***p < 0.001, ****p < 0.0001; ns, non-significant).

In order to determine the potential of *L. tarentolae* co-expressing PpSP15 and PsSP9 adjuvanted with CpG in providing protection against *L. major* infection and to characterize the type of induced immune responses, cytokines including IFN-γ, IL-4, IL-10, and IL-17 were also assessed at weeks 7 and 11 post-challenge following *in vitro* stimulation with parasite F/T. As shown in **Figure 5A**, the level of IFN-γ was detected significantly higher in the *L. tarentolae* PpSP15-T2A-PsSp9 vaccinated group (G1Lm) compared to both control groups at 7^th^ WAC. At the same time point, IL-4 production was almost similar in all three groups and respecting the production of IL-10, no significant differences were obtained between different mice groups (**Figures 5B, D**). Therefore *in vitro* stimulated splenocytes from *L. tarentolae* PpSP15-T2A-PsSP9 vaccinated mice (G1Lm), elicited significantly higher ratios of IFN-γ/IL-4 (**Figure 5C**) and IFN-γ/IL-10 (**Figure 5E**) in comparison with control groups (G2Lm and G3Lm). Besides, the production of IL-17 was substantially higher in the G1Lm group than in G3Lm while it was comparable with G2Lm (**Figure 5F**). As a result, the highest ratio of IFN-γ/IL-17 was determined for *L. tarentolae* PpSP15-T2A-PsSP9 vaccinated mice group (G1Lm) compared to G2Lm and G3Lm control groups (**Figure 5G**). This early-time cytokine profile well matched the same pre-challenge cytokine profile. *L. major* infection was then well tolerated by an increase in IFN-γ and decrease in IL-17, IL-10, and IL-4 production in G1Lm group. Interestingly, this cytokine profile at 7^th^ WAC explained the parasite load at the same time point.

Four weeks later at 11^th^ WAC, the amount of IFN-γ in the vaccinated group (G1Lm) dropped to lower levels but it was still higher than G2Lm (*p*= 0.1101) and G3Lm groups (*p*= 0.5673) (**Figure 5A**). Of note, no increase was observed at the level of IL-4 (**Figure 5B**) and IL-10 (**Figure 5D**) in G1Lm (significantly lower levels of the mentioned cytokines were detected). A remarkable decline in the level of IL-17 was also observed compared to the earlier time point (7^th^ WAC) in G1Lm and G2Lm groups (**Figure 5F**), but instead IL-17 substantially increased in G3Lm. Therefore, the highest ratios of IFN-γ/IL-4 (**Figure 5C**), IFN-γ/IL-17 (**Figure 5G**), and IFN-γ/IL-10 (**Figure 5E**) were detected in G1Lm group. This cytokine profile explained the parasite load of week 11 post-challenge with a lower but not statistically significant parasite number per cell in the G1Lm group. These results indicated that the severe pathogenicity of *L. major* infection in BALB/c mice cannot be tolerated at late time points post-infection relying on the strength of the vaccine formulation used in this study.

IFN-γ production is functionally correlated with macrophage induction of Nitric oxide (NO), a way to control parasite proliferation. Therefore, NO measurement is a functional estimation of the IFN-γ production. The amount of NO in response to *L. major* F/T antigen was measured for all mice groups at 7^th^ and 11^th^ WAC. The findings indicated that the highest level of IFN-γ in G1Lm group well matched the highest level of NO in this group at both measurement times (**Figure 5H**). Therefore the NO profile was quite in line with IFN-γ production.

**Figure 6:**
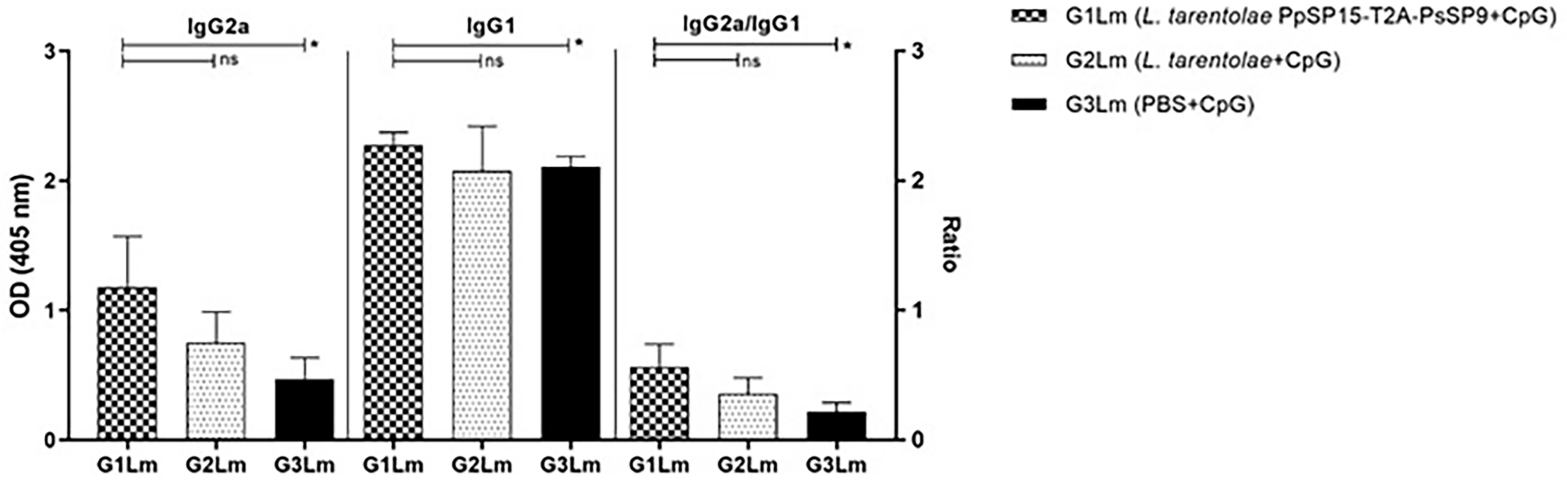
Analysis of antigen-specific antibody responses in vaccinated and control mice by ELISA post-infection with *L. major*. All mice (n=8 per group) were bled from the venous sinus to collect sera at week 11 post-challenge. The level of IgG2a, IgG1, and IgG2a/IgG1 ratio were evaluated in response to *L. major* F/T antigen. Data are representative of two independent experiments. The results are presented as mean ± SE and student t-test was used for statistical analysis (*p < 0.05; ns, non-significant).

Likewise, the antibody responses against *L. major* F/T antigen at 11 weeks post-challenge showed the highest level of IgG2a in the vaccinated group (G1Lm) and comparable amount of IgG1 in all mice groups. Therefore as shown in **Figure 6**, the ratio of IgG2a to IgG1 was comparatively higher in G1Lm than in G2Lm (*p*= 0.1675), and G3Lm (*p*= 0.0331) groups.

Collectively, these findings demonstrated a Th1 polarized response detectable up to week 7 post-infectious challenge which slightly faded at later weeks. This was illustrated in the drop down of IFN-γ/IL-4 and IFN-γ/IL-17 ratios but not in the IFN-γ/IL-10 ratio. This high amount of IL-10 could account for a suppression in Th1 potential to control *L. major* infection in BALB/c mice which was well reflected on the non-significant lower parasite load of *L. tarentolae* PpSP15-T2A-PsSP9 vaccinated group at the late weeks post-challenge.

### *L. tarentolae* PpSP15-T2A-PsSP9 Vaccination Potentially Protects *L. tropica* Infected BALB/c Mice

**Figure 7:**
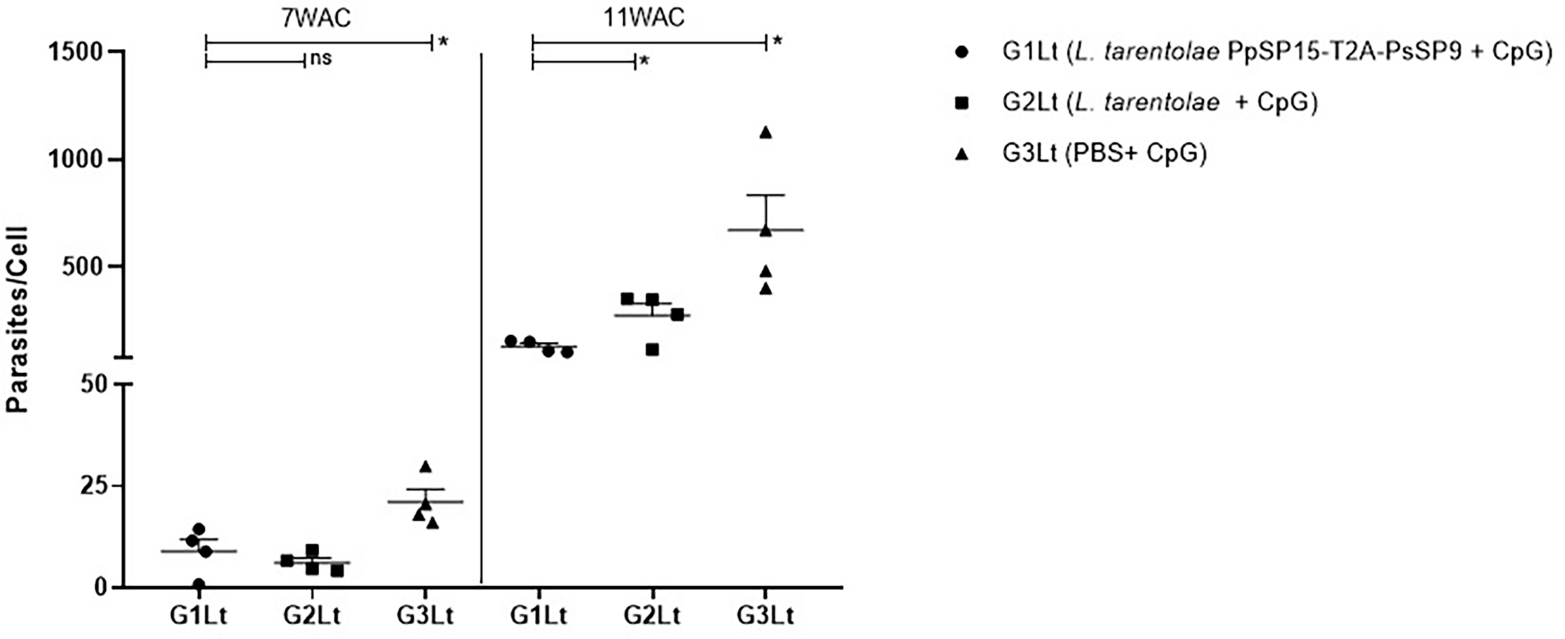
Assessment of parasite load in vaccinated and control BALB/c mice infected with *L. tropica* +SGH. Pre-treated BALB/c mice (n=28 per group) with CpG were immunized at the left footpad with *L. tarentolae* PpSP15-T2A-PsSP9 (G1), *L. tarentolae* wild-type (G2), and PBS (G3). Three weeks after the last booster, mice were challenged with *L. tropica* metacyclic promastigotes plus *Ph. sergenti* SGH in the contralateral footpad. The number of parasites per mouse lymph node cell was determined at weeks 7 and 11 post-infection by Real-Time PCR (n=4 mice per group). Results are exhibited as the mean ± SE. Data are representative of two independent experiments. Student t-test was used for statistical analysis *p< 0.05; ns, non-significant).

The other half of immunized BALB/c mice in all three groups were challenged by *L. tropica* metacyclic promastigotes along with SGH of *Ph. sergenti.* The potency of *L. tarentolae* PpSP15-T2A-PsSP9 plus CpG as a new live vaccine candidate to control *L. tropica* infection was estimated by parasite burden measurement (in this model the footpad monitoring is not measurable due to a mild footpad inflammation). As shown in **Figure 7**, at week 7 post-infection, there was no obvious difference in the number of parasites per lymph node cell between G1Lt (mean ± SE= 8.9 ± 2.9) and G2Lt (mean ± SE= 6.1 ± 1.1) groups. However, the propagation of *L. tropica* in the vaccinated group (G1Lt) versus PBS group (G3Lt) (mean ± SE= 21.1 ± 3) was significantly lower. Further evaluation of disease progression up to the week 11 after infectious challenge, revealed a significant control in the number of parasites per cell for G1Lt (mean ± SE= 130 ± 14) compared to G2Lt (mean ± SE= 274 ± 55) and G3Lt (mean ± SE= 671 ± 163) groups.

**Figure 8:**
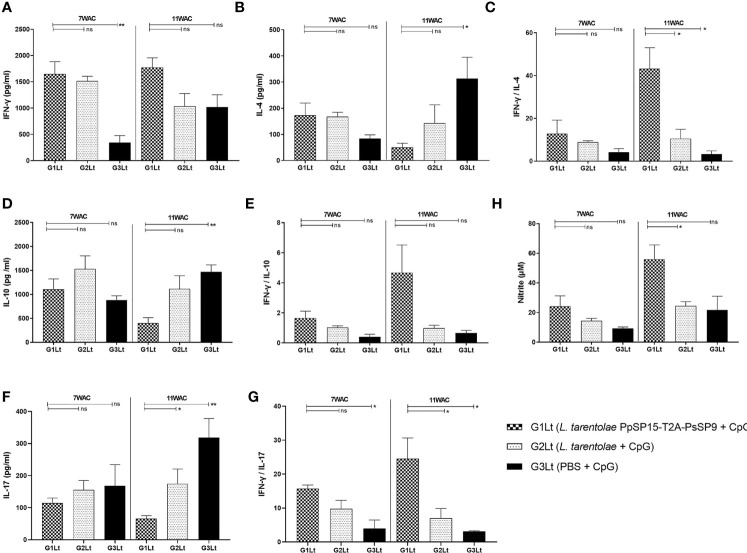
Assessment of cytokines productions in vaccinated and control groups at post-infection with *L. tropica*+ *SGH_Ph_*. *_sergenti_*. Following 7^th^ and 11^th^ weeks after challenge, all mice (n=3-4 per group) were sacrificed and splenocytes from each individual BALB/c mouse were re-stimulated by *L. tropica* F/T antigen. **(A)** IFN-γ production, **(B)** IL-4 production, **(C)** IFN-γ/IL-4 ratio, **(D)** IL-10 production, **(E)** IFN-γ/IL-10 ratio, **(F)** IL-17 production, **(G)** IFN-γ/IL-17 ratio, **(H)** Nitric oxide (NO) production. The results are presented as mean ± SE in pg/ml for cytokines or in µm for NO. Data are representative of two independent experiments. Student t-test was used for statistical analysis (*p < 0.05; **p < 0.01; ns, non-significant).

Cytokine evaluation at week 7 post-challenge indicated a remarkable increase in IFN-γ production versus pre-challenge which was comparable in G1Lt and G2Lt and significantly differed with G3Lt group (**Figure 8A**). There was no significant difference in IL-4 (**Figure 8B**), IL-10 (**Figure 8D**), and IL-17 (**Figure 8F**) production among groups. This profile was directly reflected on IFN-γ/IL-4 (**Figure 8C**), IFN-γ/IL-10 (**Figure 8E**), and IFN-γ/IL-17 (**Figure 8G**) ratios which were non-significant among all three groups but slightly higher in G1Lt. Nonetheless, the ratio of IFN-γ/IL-17 was remarkably higher in G1Lt compared to the G3Lt group. Thus, it seems that the cytokine profile at 7^th^WAC could explain the parasite load at the same time point. However, at week 11 post-challenge due to the higher level of IFN-γ (**Figure 8A**) on one hand and lower levels of IL-4 (**Figure 8B**), IL-10 (**Figure 8D**), and IL-17 (**Figure 8F**) cytokines on the other hand in G1Lt versus G2Lt and G3Lt control groups, the ratios of IFN-γ/IL-4, IFN-γ/IL-10, and IFN-γ/IL-17 in G1Lt group were significantly higher (**Figures 8C, E, G**) well explaining the parasite load profile.

**Figure 9:**
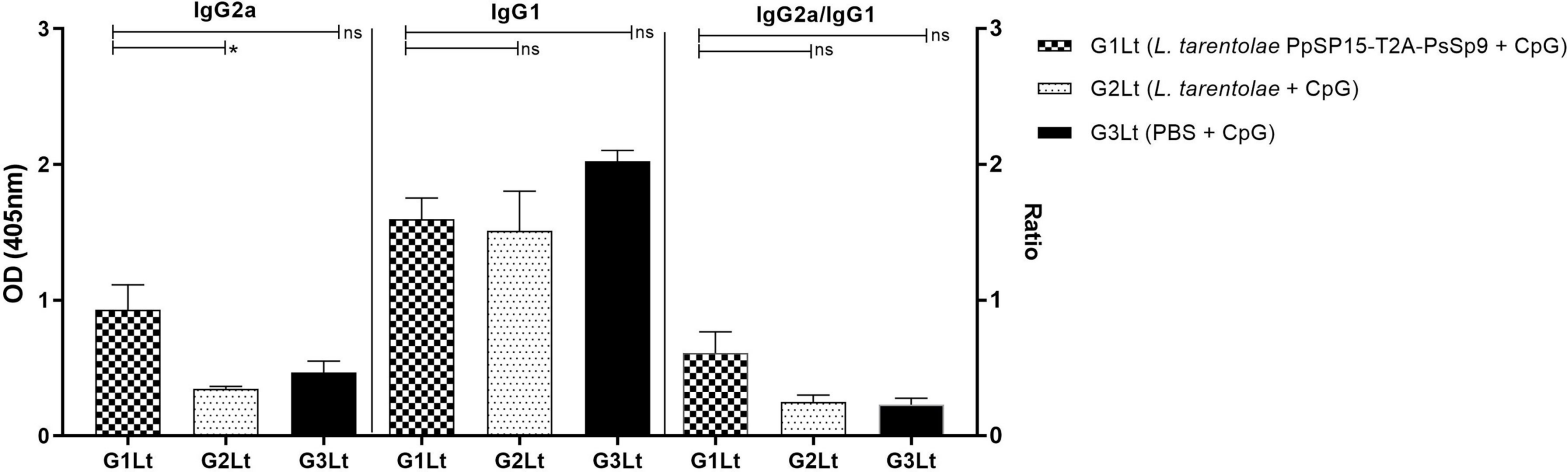
Analysis of antigen-specific antibody responses in vaccinated and control groups. The sera were collected from all mice (n=8 mice per group) at week 11 post-challenge and the levels of IgG2a, IgG1, and IgG2a/IgG1 ratio were evaluated in response to *L. tropica* F/T antigen. Data are representative of two independent experiments. The results are presented as mean ± SE and student t-test was used for statistical analysis (*P < 0.05; ns, non-significant).

Moreover, as shown in **Figure 8H**, the NO profile confirmed the higher production of IFN-γ in the *L. tarentolae* PpSP15-T2A-PsSP9 vaccinated (G1Lt) group compared to control groups (G2Lt and G3Lt) at both time points (7^th^ WAC and 11^th^ WAC) following infection. Similarly, the Th1 type skewed response at later weeks was reflected on antibody isotype. G1Lt produced higher IgG2a compared to G2Lt and G3Lt groups while the level of IgG1 was comparable within groups (**Figure 9**).

Taken together, it was concluded that the Th1 deviating potential of *L. tarentolae* PpSP15-T2A-PsSP9 formulation potentially controlled the infectious challenge by *L. tropica* up to 11 weeks post-challenge. Of note, we could point to the elevated level of IFN-γ (as ameliorating inflammatory cytokine) compared to IL-4 (as disease promoting) and IL-10 (as Th1 suppressor) cytokines during the course of infection from week 7 to 11. The IFN-γ to IL-17 ratio also increased during the infection course as a good sign of infection control. This is in contrast to *L. major* infection where all ratios dropped to lower levels at late time points leading to disease progression as expected in BALB/c model of *L. major*. A remarkable difference between *L. major* and *L. tropica* infection was a 2 fold increase in IFN-γ/IL-10 ratio following *L. tropica* infectious challenge which might best account for slowed down disease progression in BALB/c model of *L. tropica.*

## Discussion

To date, all efforts to develop protective vaccines against human leishmaniasis have failed (23). The major requirement of ideal anti-*Leishmania* vaccines is the induction of long-term Th1 immunity with significant IFN-γ production. The IFN-γ cytokine is crucial for macrophages activation in order to increase NO production and eliminate intracellular *Leishmania* parasites (32). So far, leishmanization has been known as the best way to induce long-lasting protective immunity to control *Leishmania* infection. However, this method which was based on the deliberate inoculation of live infective *L. major* parasites was discontinued due to safety concerns but indicated that live vaccines have a better chance of success (33). Therefore, live attenuated vaccines were introduced as the promising alternative for protection against *Leishmania* parasites. In parallel human nonpathogenic species such as *Leishmania tarentolae* have generated promising results as a live vaccine vector (25, 34).

Importantly, the contribution of vector’s saliva in disease progression is highly pronounced (10) and there is ample evidence that pre-exposure to the bites of uninfected sand fly induces a protective anti-parasite immunity at the bite site (11). Therefore, it seems advisable to address the potential of these components in developing effective anti-*Leishmania* vaccines. Noteworthy, the saliva composition differs between various sand fly species. This implies that the effectiveness of salivary proteins as anti-*Leishmania* vaccines could be species specific (35, 36).

With the aim of designing an effective live vaccine against *L. major* and *L. tropica*, two main pathogenic species of CL in the old world, recombinant *L. tarentolae* co-expressing PpSP15 and PsSP9 with T2A linker was generated. Herein, the T2A (thosea asigna virus 2A) peptide as a self-cleavable linker was inserted between the two salivary genes encoding vaccine candidates. These short viral-derived 2A sequences mediate self-cleavage by ribosome skipping mechanism during translation and thereby result in the discrete production of flanking proteins in almost equal amounts (37). The equal expression of the two salivary proteins was of high importance in the present study which was confirmed by Western blotting. This prophylactic vaccine candidate was then evaluated in BALB/c model of CL adjuvanted with CpG ODN as a potential Th1 immune response inducer. Importantly, CpG in combination with live pathogens enhances cell-mediated immunity against infection (38). Wu et al. have demonstrated that the administration of CpG with live *L. major* is able to prevent the lesion formation in C57BL/6 model of CL and strikingly minimized the pathology of live parasites (38). As well, Zahedifard et al. showed that immunization with *L. tarentolae* expressing cysteine proteinases (CPA/CPB/EGFP) along with PpSP15 DNA induced higher protective immunity to restrict *Leishmania* infection compared to recombinant *L. tarentolae* alone (39). It was inferred that DNA by its intrinsic properties as an immunomodulatory molecule induces potential innate immunity forcing Th1 polarization against *Leishmania* infection, therefore synthetic CpG ODNs were further co-administered with live candidates while designing vaccine against leishmaniasis (19, 40).

The aim of our study was to evaluate the protective efficacy of the recombinant *L. tarentolae* secreting sand fly salivary proteins as a dual live vaccine candidate against infectious challenges by *L. major* or *L. tropica* in the BALB/c model. The BALB/c mice are used as an animal model for both pathogens however compared to *L. major*, *L. tropica* infection progresses more slowly and skin lesions plus parasite load in organs are less pronounced (41, 42). In other words, *L. tropica* infection does not progress uncontrolled quite contrary to *L. major*. It was apparently indicated in our study that the *L. tropica* parasite load was significantly lower in number per cell compared to *L. major* detected at the same time point in the infected lymph nodes. This difference in minimal but persistent pathology of *L. tropica* infection well correlates with an apparent equilibrium in host-pathogen interaction. While Th2 response predominates against severe and progressive *L. major* infection in the BALB/c model, it does not explain the chronic infection of *L. tropica*. Instead, non-curing but non-progressive *L. tropica* infection is associated with Th1 mediated control and low levels of IL-4 (43). These substantial differences in the *L. major* and *L. tropica* experimental model were further considered in vaccine result interpretation keeping in mind that very few vaccine formulations are examined against *L. tropica* infection up to present.

In the case of *L. major* infection, following *L. tarentolae* PpSP15-T2A-PsSP9 plus CpG immunization, vaccinated BALB/c mice (G1Lm) demonstrated lower-sized lesions and significantly lower level of parasite load in draining lymph nodes when compared to control groups at early time points. However, at week 11 post-challenge, despite a remarkable control over parasite propagation, parasite load was statistically comparable between groups. In contrast, in the case of *L. tropica* infection, *L. tarentolae* PpSP15-T2A-PsSP9 plus CpG immunization significantly reduced the parasite propagation during the course of infection. This clinical manifestation well reflected the Th2 vs. Th1 dominance of the immune response in BALB/c model of CL due to *L. major*. Here we clearly indicated that the immune response was well polarized toward Th1 response before any infectious challenge. Therefore the challenge outcome was entirely *Leishmania* species dependent which was further confirmed by the cytokine profile of each infection. Cytokine profile follow-up until week 11 post-challenge indicated the highest production level of IFN-γ and NO and the lower level of IL-4 and IL-10 in recombinant *L. tarentolae* PpSP15-T2A-PsSP9 plus CpG vaccinated mice (G1Lm and G1Lt groups). In addition, the highest level of IFN-γ to IL-4 and IFN-γ to IL-10 ratios were observed in the vaccinated (G1Lm and G1Lt) groups. This cytokine profile pronounces the Th1 immune response following immunization with the new live vaccine candidate and post-challenge with both parasite species. However, the Th1 response, although mitigated the Th2 potential early after infection, faded during later weeks post *L. major* challenge smoothening rapid parasite propagation. Elikaee et al. demonstrated that an attenuated live vaccine of *L. major* parasites lacking the p27 gene (Lmp27^-^/^-^), induced a strong protective Th1 immunity which was able to control *L. major* infection up to 12 weeks post-challenge (44). Also, Salari et al. showed that a live *L. tarentolae* expressing two immunogenic antigens, LACK and KMP11, with CpG oriented the immune system toward Th1 responses which were protective against *L. major* until the 11^th^ week post-infection (40). Katebi et al. showed that co-administration of CpG ODN with recombinant *L. tarentolae* secreting PpSP15 potentially protected the vaccinated BALB/c mice against *L. major* up to week 11 post-infection (19). In other words, the greater vaccine potency to induce Th1 immune response in BALB/c model, the longer it can control the *L. major* propagation.

Quite contrary to *L. major*, *L. tropica* infection was well controlled by elevated Th1 response at later weeks post-challenge, as we observed. We could barely find similar data to compare the results due to limited existing experimental data on host immune responses to *L. tropica* vaccines. Nevertheless, our results were in line with a few previous reports. In a study by Maarouf et al., the cell-mediated immunity caused by the DNA vaccine encoding Ribosomal protein L5 was Th1 type, which controlled the progression of *L. tropica* infection for up to 6 weeks in BALB/c mice (45). Furthermore, Rostamian et al. demonstrated that vaccination with recombinant *L. tropica* stress-inducible protein-1 (LtSTI1) plus monophosphoryl lipid A (MPL), despite the low induction of immune responses (IFN-γ and IL-10), significantly reduced parasite load in BALB/c mice lymph nodes up to week 16^th^ post-challenge (46). Similar to this finding, we observed a substantial reduction in *L. tropica* parasites load level in the lymph node at week 11^th^ post-challenge. However, a more potential Th1 profile was detected. The major point is that all the *L. tropica* experiments in the literature are conducted without incorporating the considerations of sand fly transmission of parasites.

Further cytokine assays also indicated that the immunized mice with recombinant *L. tarentolae* co-expressing PpSP15 and PsSP9 plus CpG produce a remarkable amount of IL-17 prior to *Leishmania* infection. Following infectious challenges with *L. major* or *L. tropica*, the production level of IL-17 in vaccinated mice represented a decreasing trend. The IL-17 is mostly produced by Th17-CD4^+^T cells (47). The recent data pointed that *Leishmania* infection triggers the differentiation of Th17 subsets in humans and mice (48). In addition, it appears that the CpG ODNs play an important role in generating Th17 cells and the production of IL-17 (49). This cytokine by mediating the leukocytes recruitment, particularly neutrophils to the target site enhances the Th1 immune responses at the early phase (50). The critical protective role of IL-17 in the clearance of *Trypanosoma cruzi* in Chagas disease has confirmed the importance of Th17 cells in fighting with kinetoplastid protozoan parasites such as *Leishmania* (51, 52). Our findings with respect to IL-17 are in concordance with the previous report by Wu et al. (49). They found specific induction of Th17 cells and high expression of IL-17 at week two post-vaccination (early phase) with live *L. major* pathogens plus immunomodulatory molecules. According to their findings, Th17 cells and not Th1 cells were the predominant effector cell population in the early phase post-vaccine, live *L. major* plus CpG. In contrast at week 6 post-vaccination, Th1 cells were the main cell population in the vaccinated mice (49). It appears that in the early phase, both IL-17 and IFN-γ cytokines work together synergistically to promote inflammation while later the production level of IL-17 decreases due to the negative regulatory effect of IFN-γ. The relationship between Th1 and Th17 is not well established in *Leishmania* and notably *L. tropica* infection. Nonetheless, it is suggested that the Th17 cells promote Th1 immune response development (49) and to this end, CpG motifs play a substantial role in Th1/Th17 balance regulation mitigating the neutrophil recruitment by Th17. This is of paramount importance in disease progression and needs to be further clarified in respect to *L. tropica* infection. Of note, the timing of CpG administration could be a determining factor in defining the vaccine potential.

There are extensive data showing that the pre-activation of immune system with CpG improves protective immunity against infections (53). In this respect, a study by Fylen et al. demonstrated that systemic pre-administration of CpG reduced the severity of the lesion in primates following *L. major* infection (54). Indeed, CpG usage prior to live parasite inoculation activates the innate immunity to elicit earlier inflammation which leads to smaller skin lesions and accelerates the healing process (54). Furthermore, Ribes et al. showed that systemic immunostimulation with CpG three days prior to infection with *E. coli K1* enhanced mice survival by more production of pro-inflammatory cytokines (55). It is worth to mention that according to microarray analysis data (56), the effects of CpG on expressing pro-inflammatory genes are detectable only 30 min after injection. Interestingly, gene expression peaks 3hr following administration in which nearly 1000 genes are up-regulated under the regulatory control of two major inducers, IFN-γ and or TNF-α. Importantly, the level of gene expression decreased three days after injection. Considering this body of evidence about the effect of the CpG administration prior to infection on improving the vaccine potency, in this study, all mice groups received CpG ODN prior to immunization with recombinant *L. tarentolae* PpSP15-T2A-PsSP9. Contrary to the expectations raised by the background data explained here, the pre-administration of CpG was not able to enhance the vaccine potential against *L. major* [contrary to the previous report (19)] although it could still mount a higher Th1 vs Th17, Th2 and IL-10 regulatory responses. Therefore, regardless of the existing data, co-administration of CpG can further increase the vaccine effectiveness against *L. major* and more likely against *L. tropica* infection. The latter obviously needs to be further addressed.

Taken together, our findings supported the success of the newly designed multivalent live vaccine to provide protection against cutaneous leishmaniasis. To the best of our knowledge, this report represents the first evaluation of the dual vaccine candidate targeting *L. major* and *L. tropica*. The results indicated that the immunization with recombinant *L. tarentolae* co-expressing PpSP15 and PsSP9 plus CpG as a new live vaccine candidate induced Th1-mediated protection against both infectious challenges. However, the effectiveness of the vaccine needs to be further improved by additional elements namely complementary TLR ligands such as TLR2 besides TLR9 (engaged by CpG motif) and appropriate timing. Obviously, it could be inferred that a more potential Th1 response which ameliorates the disease progression post *L. major* challenge, definitely protects against *L. tropica* in BALB/c model. The overall approach is worth evaluating especially in endemic areas where different sand fly vectors frequently transmit different parasite species.

## Data Availability Statement

The original contributions presented in the study are included in the article/**Supplementary Material**. Further inquiries can be directed to the corresponding authors.

## Ethics Statement

The animal study was reviewed and approved by Institutional Animal Care and Research Advisory Committee of Pasteur Institute of Iran (ethical code: IR.RII.REC.1394.0201.6417).

## Author Contributions

Conceptualization: SR and NS; Data curation: ML, NS, and SR; Formal analysis: ML; Funding acquisition: SR and YM; Investigation: ML, NS, SR, TT, EG, HS, and SH; Methodology: ML, TT, EG, and NS; Project administration: SR and NS; Resources: SR; Supervision, Validation, Visualization: NS and SR; Writing – original draft: ML; Writing – review and editing: NS, TT, and SR. All authors contributed to the article and approved the submitted version.

## Funding

This work was supported by Pasteur Institute of Iran (grant number 1106), Iran National Science Foundation (grant number 940007), Zanjan University of Medical Science (grant number A-12-190-26), and European Union’s Horizon 2020 research and innovation program under the Marie Skłodowska Curie Actions (grant number 778298).

## Conflict of Interest

The authors declare that the research was conducted in the absence of any commercial or financial relationships that could be construed as a potential conflict of interest.

## Publisher’s Note

All claims expressed in this article are solely those of the authors and do not necessarily represent those of their affiliated organizations, or those of the publisher, the editors and the reviewers. Any product that may be evaluated in this article, or claim that may be made by its manufacturer, is not guaranteed or endorsed by the publisher.

## Abbreviations

CL, cutaneous leishmaniasis; ZCL, zoonotic cutaneous leishmaniasis, ACL, anthroponotic cutaneous leishmaniasis; *Ph. papatasi, Phlebotomus papatasi*; SGH, salivary gland homogenate; SDS, sodium dodecyl sulfate; FCS, fetal calf sera; BSA, bovine serum albumin; DAB, diaminobenzidine; BCA, bicinchoninic acid; ODN, oligodeoxynucleotide; RT-PCR, reverse-transcription PCR; S.C., subcutaneously; pLNs, popliteal lymph nodes; gDNA, genomic DNA; GAPDH, glyceraldehyde 3-phosphate dehydrogenase; Con A, concanavalin A; F/T, freeze-thawed; NO, nitric oxide; NED, naphthyl ethylenediamine; T2A, thosea asigna virus 2A peptide; LtSTI1, *L. tropica* stress-inducible protein-1; MPL, monophosphoryl lipid A.
