# Supplementary figures and images for: Leishmania tarentolae as Potential Live Vaccine Co-Expressing Distinct Salivary Gland Proteins Against Experimental Cutaneous Leishmaniasis in BALB/c Mice Model
Source: Front Immunol. 2022 Jun 10;13:895234. doi: 10.3389/fimmu.2022.895234 (PMC9226313; doi:10.3389/fimmu.2022.895234)

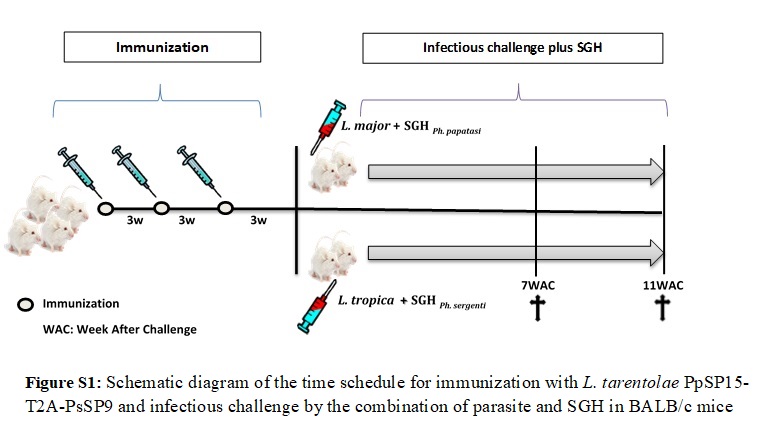

Supplement: Supplementary file 1 [file Image_1.jpeg]
